# Forging an interdisciplinary lens for understanding community digital archives of South Asian diaspora

**DOI:** 10.3389/fsoc.2025.1450641

**Published:** 2025-06-05

**Authors:** Sharika Parmar

**Affiliations:** Humanities, FLAME University, Pune, India

**Keywords:** community archives, South Asia, migration, interdisciplinary, sociology, history, anthropology, digital humanities

## Abstract

Different communities have begun archiving their own experiences and histories as a way to reclaim narratives and contend with their own identities and belonging. As the types of archives diversify and the role of digital technologies in archival practices expands, we are increasingly seeing digital community archival efforts. While archives have been key for carrying out research in the social sciences and the humanities, and are periodically found as topics of study in disciplinary subfields concerning themselves with the digital, there is little research on the specific subject of community digital archives. In this essay, I argue that community digital archives are important objects of sociological and historical inquiry. I discuss two community digital archives of the South Asian diaspora – the South Asian American Digital Archive (SAADA) and 1947 Partition Archive. I show that they offer insights into migration histories and notions of belonging and identity of South Asian diaspora not only through the digital records they produce, but also through how they operate using digital connectivity. I demonstrate that an interdisciplinary lens is key for critically engaging with these archives.

## 1 Community archiving and the digital

With the turn of the twenty-first century, community-based archiving projects have proliferated^1^ and are challenging traditional ideas about archival theory and praxis (Bastian and Flinn, 2020, xix). From home videos, images from personal photo albums and audio-visual testimonials of displacement, to musical traditions among pastoral and agrarian communities, objects of archival endeavors are diversifying. Collections are being built through volunteered material, crowdsourcing efforts, and collaborative engagements.^2^ They are also being shared varyingly through websites, Facebook and Instagram pages, and digital platforms designed to enable diverse archival practices.^3^ These new ways of archiving unsettle notions of who gets to archive, how it should be done, and what constitutes a record.

As the practice of archiving no longer remains confined to the state (whether the colonial or the nation state), the norms of who gets to archive are being reworked. Different systems of archiving that push against imperial antiquarian and colonial logics are now visible. An alternative, anti-colonial archiving process is possible when we make space for different understandings of collecting and acknowledge networks of relationships that enable it (Christen and Anderson, 2019, 87). We are seeing that different practices of collecting for archives are gaining legitimacy, particularly in community archival projects. Archival records and collections are being assembled not only by professional archivists, but also by volunteers and ‘story scholars’ or citizen historians.^4^ Records and collections are being consciously created by or co-created with people whom the record is about, through continued engagement with (and by) community members. The changing collection practices, as well as the diversification in the form and content of archival records is accompanied by an increasing role of digital technology in archival practice.

Digital technologies inflect processes of archival record creation as well as dissemination among these community archives. Changing role of digital technologies in archival practice affects who can be part of the process of archiving and how archival objects are collected and created. Practices such as crowdsourcing testimonies, photographs and even annotations to archival objects are enabled by digital recording devices (such as video recorders, mobile phone cameras, portable microphones) and connectivity and storage infrastructure (internet and cloud). This leads to creation of new types of records (such as the digitized photograph or audio-visual oral history testimony), prompting us to rework definitions of what constitutes an archival record. Technologies of archiving shape not only what is archived but also all future interactions with that archived content (Derrida and Prenowitz, 1995, 16). We see experiences of different communities increasingly being not only archived but also shared through digital mediums (such as websites, Facebook, Instagram, and Youtube pages of archives). In light of this, we may proceed to ask, how are digital technologies shaping archivable content within community archives, and how do they affect the access to this archived material?

Taking community digital archives as an object of study in the social sciences and humanities allows us to inquire into such facets of archival records and access to these records. It allows questioning of the conventional understanding of archives and the archival process. Archives have not only been pertinent for methodological repertoire of the disciplines of Historical Sociology, History and Anthropology, but are increasingly found as topics of study in most disciplinary subfields of Sociology, History and Anthropology that concern themselves with the digital (See Lupton, 2015; Crymble, 2021; Milligan, 2022). However, there remains much to be explored about the specific form of community digital archives. Research on community digital archives is currently limited and is being attempted in disciplinary silos. This prevents them not only from being understood comprehensively as objects of inquiry in their own right, but also impedes development of research that is directly relevant to sociological discourses and themes. In this essay, I assert that community digital archives are important objects of sociological and historical inquiry and that an interdisciplinary approach is required for analyzing them. I demonstrate this through reflections on the South Asian American Digital Archive (SAADA) and 1947 Partition Archive.

## 2 Reflections on two community digital archives of South Asian diaspora

The two archives discussed here are the South Asian American Digital Archive (SAADA) and the 1947 Partition Archive. They are illustrative of digital archival efforts that seek to archive experiences of South Asian diaspora of the United States of America and survivors of the Partition of India and Pakistan, “one of the largest mass migrations in human history” that violently displaced over 14 million people (Perkins, 1947). These two archives are regarded as community archives because their records are created by or co-created with the people about whom they seek to inform us about. Conceptions of community archives are broad and constantly evolving, and have often been synonymous with endeavors that describe themselves using different terms, such as ‘oral history project’, ‘community history project’, ‘community memory project’, and coalesce around locality, shared beliefs or common purpose (Flinn, 2007, 152–3). As use of this term has grown, a crucial characteristic of community archives which is highlighted is that their collections, records or objects are created or collected and held by the community. They include a range of archival endeavors that often go unrecognized by traditional or institutional archives and result from people’s expression of a certain identity constructed with or in opposition to certain groups (Bastian and Flinn, 2020, xx-xxi). The records of SAADA and the 1947 Partition Archive are created in collaboration with the South Asian populations that either migrated to the United States of America or survived the violent displacement of Partition, and this co-creation is enabled by digital technology.

SAADA’s collections are created by digitizing materials loaned by various individuals, families and organizations of the South Asian diaspora in the United States of America or produced by members of the diaspora for the archive. The 1947 Partition Archive’s oral history interview collection is built through consensual interaction with people who experienced the event of the Partition of India and Pakistan, as they share their memories with the archive’s volunteer interviewers called Story Scholars. Their archival material is digital, comprising digitized photographs, oral history interviews and other audio-visual recordings made in the past (such as home videos), as well as remixed content and original content like songs and podcasts composed for the explicit purpose of recording diasporic experiences and expressing identities.^5^ The diverse media that make up these two archives are created through digital technologies of recording and processes such as digitization. Co-created with migrants who trace their origins to South Asia, SAADA and the 1947 Partition Archive are community digital archives of diverse South Asian diaspora.

They are attempts to find space in the American historical national narrative and the mainstream history of the Partition, enabled by the digital. The proposed aim of SAADA is to ensure “that South Asian Americans are recognized as an essential part of the American story”,^6^ and of the 1947 Partition Archive is building or “institutionalizing the people’s history of Partition”.^7^ This reflects the desire to make South Asian Americans visible in the history of the United States of America, and the wish to highlight a non-state or bottom up perspective of the migration resulting from the Partition. These archival efforts seek to record historical migrations as defined and understood by the people who experienced them, making perspectives of the experience of migrations and their afterlives visible through the digital space. The audio interview of Khataw (n.d.) from the ‘First Days’ collection of SAADA (SAADA, accessed 2024). In this interview he shares the experience of his first day in the United States, recollecting the earliest moments of arriving in Fayetteville, Arkansas as a twenty-one year old in 1980. While he had moved away from Karachi, Pakistan (where he was born) at the age of eight and had lived in Hong Kong and England before migrating to the USA, he found moving to the USA unique. This was not because of a “culture shock” he clarifies, but because of the intense loneliness and helplessness he felt on arrival. He describes that he experienced homesickness for the first time because he was away from his parents, siblings and friends. “You do not feel like you fit in” he recounts, expressing the feeling that he did not feel like he belonged there. He states that his immediate response was to look for “desis,” a term used by South Asians for one another. He recollects that he did not have “anyone to count on” or “ask for advice.” Ali Khataw’s audio interview from SAADA’s archival collections highlights his concerns and emotional turmoil, which despite being short-lived (he states things felt different within just a month after arrival) was extremely intense and remembered sharply even four decades later, giving us insights into the earliest experiences of a young South Asian migrant in the United States in the ‘80s.

Such types of records about migrant experiences of South Asian diaspora which foreground the perspective of someone from the community did not exist in traditional archives like national archives, and it is rare to find them in such institutional archives even now. Also consider the interview summary or “story summary” of Mai Taji’s oral history interview from the 1947 Partition Archive’s collection, made accessible through a digital exhibit hosted on the website of Stanford University Libraries, which is a brief summary of their interview penned by ‘story scholar’ Hassan (2016). It is a description which also forms a metadata entry for this record, giving a quick overview of the topics themes the record delves into, such as everyday life in agrarian areas of Amritsar district of pre-Partition Punjab and loss of home experienced due to the forced displacement. The oral history audio interview of Ali Khataw and the story summary of Mai Taji’s interview are born digital records. The combination of content and form together is novel. Thus, new kinds of archival objects and collections are being created and are gaining legitimacy, expanding and evolving the definitions of what is considered a record. Moreover, in these two archives majority of the records are created as they are acquired: digitization of photographs, creating digital copies of old videos, and recording audio or video interviews, which in the case of the 1947 Partition Archive are collected with the help of citizen historians (‘story scholars’) who interview Partition survivors and upload the born-digital record of the testimonies to the archive’s servers. Digital technology allows for this kind of public crowdsourced collection of testimony through personal recording devices like mobile phone cameras and portable mics, allowing these archives to source their records from the people they seek to represent and enabling co-creation.

These archives also offer insights into migration histories and notions of belonging and identity through how they operate using digital connectivity. The two archives use the form of Facebook pages, Youtube channels, Instagram posts and email newsletters, apart from blogs and websites, to reach out to prospective contributors. The contributors also make up a key share of their audience. Muller views these audiences as “prosumer(s)” who consume and produce content at the same time (Müller, 2017, 17). The 1947 Partition Archive also shares excerpts of its materials on youtube, including testimonials by story scholars. Farhana Afroz, a software engineer by profession with a family history of migration across India, Pakistan and Bangladesh and a volunteer interviewer with archive shares her views in the Volunteer Clips filmed and curated by the archive and shared on Youtube (Afroz, 2011). Afroz is among several “story scholars” who seek and record oral history testimonies of Partition survivors. “Story scholars” or volunteer interviewers associated with the archive are often descendants. Experiences and stories of Partition traveled through intergenerational networks, kept alive not lost to history through people across generations and were not stored in not stored in any national archive until very recently. Evidently, these archives function through links to these communities. They tap into a sense of belonging (or unbelonging) and a sense of identity or personal history for creating their archival collections, and use digital spaces not only to popularize their endeavor but also to enable it and sustain it.

These digital archival records give insight into experiences of events of migration and the experiences of living, working and (re)building lives after it. These digital archival collections seek to create space for hitherto unheard voices across space and time. The functioning of these digital archives nudge us to consider them also as instances of users of digital technologies increasingly becoming observers and documenters of their own lives. How does this impact the way communities remember their pasts? How does this affect archival practice? Does it alter the contours of access to archival records? Researching them also opens up a space to contemplate how, particularly, the digital spurs new possibilities of remembering and expressions of post-memory and belonging. How do diasporas use digital technologies to not only maintain social networks but how those networks are reflected in archival and historical practices. What are the spaces created through digital remembering, networks and histories (offline and online) it builds upon to emerge and continue? These objects of inquiry that are underpinned by questions about researching the digital and require us to draw from disciplines across the social sciences and humanities.

## 3 Finding an interdisciplinary path for understanding community digital archives

How should we begin trying to understand these new kinds of records and relationships that traverse worlds enmeshed with the digital, and which might give us insight into the socio-historical phenomenon of migration? As we seek to grapple with experiences of migration through sources or data that was not available before, what research approaches do we require and which discipline(s) do they draw from? To understand its object of inquiry this essay draws upon discourses and methods from different disciplines across the humanities and social sciences. Such an approach, of choosing techniques, tools and/or theories from multiple disciplines for the purpose of seeking answers for the research problem at hand, is a key route to and characteristic of interdisciplinarity (Remesh and Kumar, 2024, 10). In this essay, I draw from a repertoire of historical, anthropological and sociological methods and discourses to analyze the multiple facets of the creation and consequences of community digital archives.

In attempting to comprehend records of community digital archives, it is crucial to engage with historical methods of reading archival sources. Historical method emphasizes that sources (any archival material) be examined in their context. The awareness of how a source comes into being must be threaded into the analysis of that source or record as one attempts to understand a historical experience based on it. Why was it created, under what circumstances, when, and by whom are crucial to gleaning any understanding from a record, whether physical or digital. The reading of any digital archival material too must carry in it an attention to the context of that material. Thus, we need to approach what these archives offer with such a methodological repertoire. We must critically engage with the context of the creation of their digital archival material, asking why, when and by whom they were (co-)created and what patterns might be visible across such digital archival endeavors. SAADA and 1947 Partition Archive consciously aim to claim space in the historical records of a nation or event. This context and purpose must inform our readings and analyses of these archives and their records. Therefore, our readings of records SAADA and 1947 Partition Archive must be informed by the conscious purpose of these archives to claim space in archival and historical records of a nation or an event.

The context of production of an archive itself is worth studying. Archives have had a double relationship with disciplines within the social sciences and humanities. They form sources of information or data, as well as objects of inquiry. Consider anthropological work such as *Along The Archival Grain* in which Ann Stoler studies colonial records housed in the Dutch national archive. Her work shows that the documents can be examined to reveal not only information about colonization of Indonesia by the Dutch, but also how the Dutch colonial state came into being. Grounded in anthropology and history, her study of those records brings out the anxieties, uncertainties and fears of empire in practice. At the same time its ethnographic focus on the creation of the documents underscores how the process of production of those documents helped constitute the administrative apparatus and practices of the Dutch colonial state (Stoler, 2010). Drawing upon Anthropological work with archives that foregrounds the context of production of archives and creation of archival records helps us see the two community digital archives discussed above as more than merely a repository of evidence about migrations. We must also view them as a consequence of the migrations they speak of. They are an afterlife of the migrations and displacements that they reveal information about, and are expressions of migrant identity and diasporic experiences in the contemporary.

The context of production of archives also relates to notions of power, selection and incompleteness of archives. Theories from critical archival studies focus analysis on how power permeates across the “context of record creation, of archival functions, of the formation of archival institutions, of archival outreach and use and advocacy, of who becomes archivists and how and why” (Caswell et al., 2017, 3). There is a process of selection of materials always underway in archives which, as practising archivists and historians point out, shapes the histories that are told and the narratives and knowledge that are produced (Caswell et al., 2017, 2; Guerrero, 2022). An archive, it seems, is never complete, neither in the sense of collecting all possible information (Staveley, 2024) nor all perspectives. In our analyses we need to account for records that have not been digitized or which were not archived hence do not leave digital traces (Milligan, 2022, 6; Markham, 2013, 439; Zaagsma, 2022, 830). The cautious call that a more representative archive does not reflect a complete record or contain more straightforward information resounds across the humanities and social sciences. Thus, we must be cognizant that SAADA and the 1947 Partition Archive present newer or different facets of the experience of migration. We must not mistake them for an “authentic” view, or a complete synthesis of the realities around the migration of those communities. They are a series of perspectives yet incomplete and affected by a prioritization of perspectives, and they must be studied as such.

To contend with how digital archiving is being used to express one’s identity and claim space socially or politically it is imperative to look at perspectives from sociology and digital sociology. Sociology is concerned deeply with the subject of identities. Sociologists also examine the impact of digital technologies on everyday life and social relations, and research social interactions that take place digitally. They have gone on to identify and typologize the sub-field of digital sociology. Digital sociology extends itself across a range of issues, from employing digital tools for sociological research to studying how people use the digital for self expression and community (Fussey and Roth, 2020). Through these perspectives, it becomes possible to understand how the digital and the social are imbricated. There is an increasing role of technology in shaping social relationships and concepts such as selfhood and space, and a need to explore how use of technology is structured through categories such as class, gender, location, age, and race among others (Lupton, 2015, 188). Careful consideration of these themes is crucial for understanding the nuances of how shared experiences of communities become a point of identity assertion digitally, through digital community archives.

While researching social interactions that take place digitally is a sociological concern, studying the social implications of digital technologies is a “cross-disciplinary endeavour.” (Fussey and Roth, 2020, 671). If inherent in the process of recording and remembering is a destruction and forgetting (Derrida and Prenowitz, 1995), and if the nature of the historical endeavor is selective (Carr, 1961), then we might ask: what is erased in constructing social histories from these digital archival records? And if perceptions of digital media are not inert but mediated (Geismar, 2018, 88), then we might ask, furthermore: how are objects of community digital archives perceived by different audiences and what meaning do they hold for these audiences? Multiple disciplinary discourses and methodological paths direct us to recognize the curation, incompleteness and silences of archives, as well as the conceptualizations of digital mediums and interactions through them. It is these diverse discourses and methodologies, then, that must guide us in crafting an approach to researching community digital archives.

## 4 Conclusion

In this essay, through reflections on the South Asian American Digital Archive (SAADA) and 1947 Partition Archive, I argue that community digital archives form important objects of sociological and historical inquiry. While not a typical subject of study in itself within Sociology or History, the process of creation of community digital archives and the impacts they have are of sociological and historical concern. Taking community digital archives as an object of study in the social sciences and humanities allows us to ask new questions. We might ask how are digital technologies shaping archivable content within community archives and access to archived material? As users of digital technologies increasingly become observers and documenters of their own lives, how does this impact the way communities remember their pasts? The social nature of these archives, in the way they are produced through links to the community, or the way that they make their presence felt through social media, is an intriguing point of social science research. We might ask: how do diasporas use digital technologies to not only maintain social networks, but also how are those networks reflected in archival and historical practices? The digital can spur new possibilities of remembering, and expressions of belonging and ownership of diasporic identity. We look to spaces of digital remembering with the recognition that society and technology shape each other, and ask: how is the use of digital technology shaped by the experience of migration or diasporic identity?

There are insights to be gained across disciplines from accessing community digital archives of diaspora communities, especially about experiences of living, working and rebuilding of lives after a migration. The discussion highlights that these community digital archives are a way to claim space in the historical record, done using digital space. The records and collection within these two archives are an assertion of experiences and identities of the people whom they speak about. These community digital archives are then sustained by people’s need for identity and expression, which is oftentimes framed by their collective historical experiences. Through studying community digital archives of diaspora communities, one can trace how identities of individuals and communities are informed through experiences of migration. They are a study in how one’s identity as a migrant influences one’s self-perception and social world, their negotiations in interactions with members of the community and outsiders, their view and record-keeping of their histories, and their perception of themselves and their personal pasts. These archives not only house impressions of afterlives of migrations but themselves constitute afterlives in the way that they function through diasporic networks consequent to those migrations. Archiving becomes a way to reclaim narratives about specific histories as well as contend with identities and belongingness.

Community digital archives are an object of inquiry best approached interdisciplinarily, drawing from across disciplines such as sociology, history, anthropology and critical archival studies. Critical perspectives from the point of view of sociologists, anthropologists, historians and archivists are fundamental to comprehending community digital archives. The reflexive engagement of historians with their sources, sociologists on the digital context, practical delineations of archivists on their profession, and philosophical reflections on archives and historical endeavors (such as those by Jacques Derrida and Walter Benjamin) form the foundation for viewing and understanding community digital archives Benjamin, 2006; Derrida and Prenowitz, 1995). When we bring together critical perspectives toward archives and digitality from sociology, history, anthropology and critical archival studies, we are able to study community digital archives critically. From the necessity of examining a source (or data) in the context of its production and circulation, the incompleteness and silences in archival endeavors to the continuities that pervade digital technology use, they provide a theoretical framework for understanding community digital archival efforts. This combination of disciplinary perspectives also allows us to expand the subject matter of the disciplines of History and Sociology. More questions of interest to the humanities and social sciences arise when we contemplate community digital archives from an interdisciplinary lens.

Questioning the entities here described as community digital archival through the paradigms of these disciplines also allows a reflection on the methodologies and future of these disciplines, which expands the options for current and future researchers working in various fields of study. As new objects of study emerge and existing methods are required to be adapted again to them, an uneasiness appears in the praxis and theory of disciplines and it stirs fundamental disciplinary discourses. The digital presents the social sciences and humanities with new objects of inquiry (such as community digital archives) and dilemmas of method. Digital humanities provides a conducive space for critically contemplating community digital archives as an object of inquiry. Rooted in deep humanistic questions of context, sources, method and meaning (Staveley, 2024), it is a field that concerns itself immensely with archives. It uses methods and critiques from across disciplines in the social sciences, humanities and computational sciences to raise newer questions. It is a location from which one may critically engage with community digital archives, allowing for a greater appreciation of diasporic community digital archives.

## Data Availability

The original contributions presented in the study are included in the article/supplementary material, further inquiries can be directed to the corresponding author.

## Footnotes

^1^See South Asian American Digital Archive (https://www.saada.org/), 1947 Partition Archive (https://in.1947partitionarchive.org/), Archive at Rupayan Sansthan (https://www.sahapedia.org/understanding-rajasthan-through-its-agrarian-zones; https://eap.bl.uk/collection/EAP1153-1/search).

^2^For example, see Soboicar (https://www.citizensarchiveofindia.org/project/Soboicar), Indian Memory Project: Tracing the History and Identity of the Indian Subcontinent Via Images Found in Personal Archives (https://www.indianmemoryproject.com/about/).

^3^See The Citizens Archive of Pakistan (https://citizensarchive.org/), 1947 Partition Archive Facebook Page (https://www.facebook.com/1947PartitionArchive/), Mukurtu platform (https://www.neh.gov/article/mukurtu-digital-platform-does-more-manage-content).

^4^See https://in.1947partitionarchive.org/storyscholars/, https://in.1947partitionarchive.org/collect_stories, https://ncph.org/history-at-work/where-are-the-citizen-historians/, https://www.c2dh.uni.lu/projects/public-history-new-citizen-science-past-phacs, https://www.uni.lu/c2dh-en/research-projects/phacs/, https://aaslh.org/5-citizen-history-projects-you-should-know-about-part-2/.

^5^https://www.saada.org/browse/types, https://in.1947partitionarchive.org/collections.

^6^ https://www.saada.org/about

^7^ https://in.1947partitionarchive.org/mission
